# FTO contributes to hepatic metabolism regulation through regulation of leptin action and STAT3 signalling in liver

**DOI:** 10.1186/1478-811X-12-4

**Published:** 2014-01-10

**Authors:** Amélie Bravard, Guillaume Vial, Marie-Agnès Chauvin, Yves Rouillé, Bernard Bailleul, Hubert Vidal, Jennifer Rieusset

**Affiliations:** 1 INSERM U-1060, Laboratoire CarMeN, Université Lyon 1, INRA 1235, INSA de Lyon, Facultés de médecine Charles Mérieux, Lyon-Sud, Oullins, France; 2 Inserm U1019, CNRS UMR8204, Center for Infection & Immunity of Lille (CIIL), Institut Pasteur de Lille, Université Lille Nord de France, Lille, France; 3 INSERM UMR-1011, Université Lille 2, Institut Pasteur de Lille, Lille, France; 4 UMR INSERM U1060, Faculté de médecine Lyon-Sud, 165 chemin du grand Revoyet, BP12 69921, Oullins cedex, France

**Keywords:** FTO, Liver, Glucose homeostasis, Mitochondria, Leptin receptor-STAT3 pathways

## Abstract

**Background:**

The fat mass and obesity associated (FTO) gene is related to obesity and type 2 diabetes, but its function is still largely unknown. A link between leptin receptor-signal transducers and activators of transcription 3 (LepR-STAT3) signalling pathway and FTO was recently suggested in the hypothalamus. Because of the presence of FTO in liver and the role of LepR-STAT3 in the control of hepatic metabolism, we investigated both in vitro and in vivo the potential interrelationship between FTO and LepR-STAT3 signalling pathway in liver and the impact of FTO overexpression on leptin action and glucose homeostasis in liver of mice.

**Results:**

We found that FTO protein expression is regulated by both leptin and IL-6, concomitantly to an induction of STAT3 tyrosine phosphorylation, in leptin receptor (LepRb) expressing HuH7 cells. In addition, FTO overexpression in vitro altered both leptin-induced Y705 and S727 STAT3 phosphorylation, leading to dysregulation of glucose-6-phosphatase (G6P) expression and mitochondrial density, respectively. In vivo, liver specific FTO overexpression in mice induced a reducetion of Y705 phosphorylation of STAT3 in nuclear fraction, associated with reduced SOCS3 and LepR mRNA levels and with an increased G6P expression. Interestingly, FTO overexpression also induced S727 STAT3 phosphorylation in liver mitochondria, resulting in an increase of mitochondria function and density. Altogether, these data indicate that FTO promotes mitochondrial recruitment of STAT3 to the detriment of its nuclear localization, affecting in turn oxidative metabolism and the expression of leptin-targeted genes. Interestingly, these effects were associated in mice with alterations of leptin action and hyperleptinemia, as well as hyperglycemia, hyperinsulinemia and glucose intolerance.

**Conclusions:**

Altogether, these data point a novel regulatory loop between FTO and leptin-STAT3 signalling pathways in liver cells, and highlight a new role of FTO in the regulation of hepatic leptin action and glucose metabolism.

## Background

Obesity and type 2 diabetes have reached epidemic proportions worldwide. Besides environmental factors, genetic factors largely contribute to the development of these pathologies. Among the susceptibility genes, the “fat mass and obesity associated” (FTO) gene may be one of the molecular determinants linking both pathologies [1]. Single-nucleotide polymorphisms identified in the gene appear to affect FTO expression levels, since FTO transcripts containing the risk allele were more abundant than those containing the wild-type allele [2]. In agreement, we recently found an increased FTO expression in both human skeletal muscle [3] and subcutaneous adipose tissue [4] during type 2 diabetes. Moreover, genetic modulations of FTO in mice showed that overexpression results in obesity [5], while inactivation of the gene is protective [6].

Leptin is a multifunctional hormone produced mainly by adipose tissue, and involved in the regulation of food intake and energy homeostasis through its central actions [7]. Athough leptin receptors (LepR) are abundantly expressed in the brain, they are also present in peripheral tissues, indicating that leptin can exert peripheral actions [8,9]. The long form receptor (LepRb) regulates intracellular signalling cascades including the JAK-STAT pathway. JAK-mediated phosphorylation of STAT3 on tyrosine (Y705) induced its relocation to nucleus, where, as a dimer, it binds to specific DNA sequences and promotes gene expression. Interestingly, it was recently demonstrated that STAT3 could also be phosphorylated on serine residue (S727), mediating the recruitment of STAT3 to mitochondria where it promotes oxydative metabolism [10,11].

FTO is expressed in many tissues with high abundance in hypothalamus and liver [12-14]. Whereas confusing data are found concerning the hypothalamic regulation of FTO expression by nutritional status [12,13,15,16], one intriguing result is that LepRb-STAT3 signalling pathway could be implicated in FTO regulation by energy restriction in hypothalamus [12]. In addition, FTO overexpression induced the mRNA levels of STAT3 in the arcuate nucleus of rat hypothalamus [17]. Consequently, these data suggest a possible cross-talk between FTO and the LepRb-STAT3 signalling pathway, which could potentially occur in other tissues, especially in liver where it might play a role in metabolic control. Indeed, STAT3 has been involved in the regulation of hepatic gluconeogenesis [18] by repressing G6P gene expression [19]. Although, very few studies focused on FTO in liver, it was shown that FTO mRNA is either not altered by energy restriction in rat liver [12] or up-regulated by fasting in mice [20] and chicken [21], although FTO protein level appears not modified by fasting [16]. We therefore concidered that it might be of importance to better understand the potential link between FTO and LepRb-STAT3 signalling pathway in the control of hepatic metabolism.

To this aim, we investigated in vitro the potential relationships between FTO and the LepRb-STAT3 signalling pathway using human hepatic HuH7 cells, and, we studied the impact of in vivo FTO overexpression in mice liver on leptin signalling and glucose homeostasis. Our study revealed a novel regulatory loop between FTO and the LepRb-STAT3 pathways and demonstrated a new role of FTO in the regulation of hepatic leptin action and glucose metabolism.

## Results

### FTO expression is regulated by the LepRb-STAT3 signalling pathway in HuH7 cells

**Figure 1:**
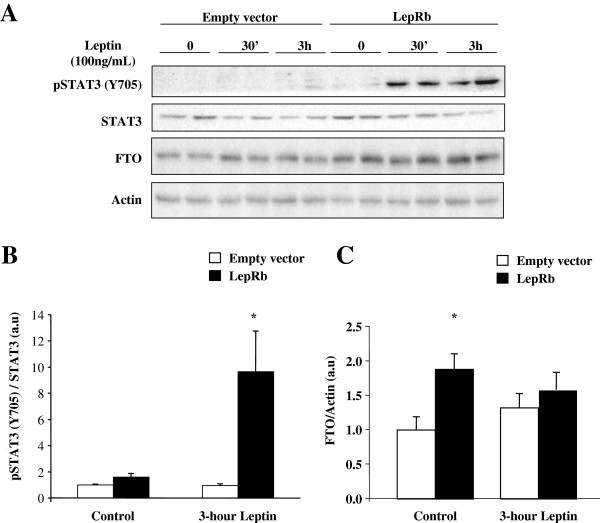
**Leptin regulates STAT3 signalling and FTO expression in HuH-7 cells.** HuH7 cells were transfecting with pcDNA3-LepRb and empty pcDNA3 vectors for 36 hours. Cells were then serum starved for 16 hours before leptin stimulation (100 ng/mL) during the indicated periods. **A)** Representative Western Blots of pSTAT3(Y705) and total STAT3, as well as FTO and actin proteins, in HuH-7 cells expressing LepRb treated with leptin (100 ng/mL). **B-C)** Quantitative analysis of both Y705 phosphorylation of STAT3 **B)** and FTO expression **C)** in 3-hour leptin treated transfected HuH7 cells, showing that **B)** leptin increases Y705 phosphorylation of STAT3 in LepRb expressing HuH7 cells, and that **C)** the overexpression of LepRb induces FTO protein levels. Data are means ± SEM (n = 6/group). *p < 0.05 compared to control transfected cells. LepRb: leptin receptor; STAT3: signal transducer and activator of transcription 3; FTO: fat-mass and obesity associated gene.

**Figure 2:**
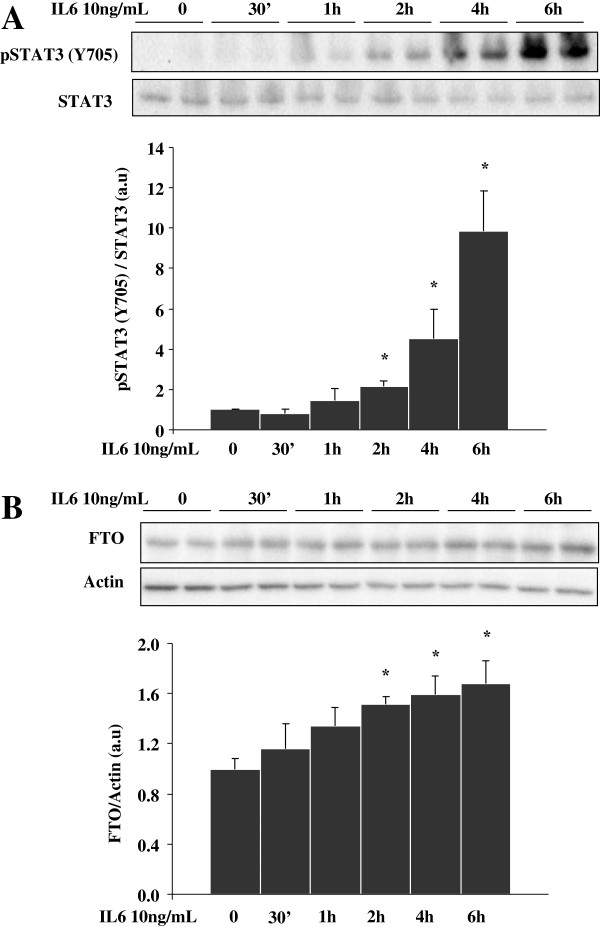
**IL-6 regulates STAT3 signaling and FTO expression in HuH7 cells.** HuH7 cells were depleted for 16 hours of serum and treated with IL-6 (10 ng/ml) during different times. **A)** Representative Western blots and quantitative analysis of Y705 phosphorylation of STAT3 in HuH-7 cells treated with IL-6 (10 ng/ml), showing that IL-6 stimulates tyrosine phosphorylation of STA3 in a time-dependant manner in HuH7 cells expressing LepRb. **B)** Representative Western blots and quantitative analysis of FTO expression in HuH7 cells treated with IL-6, showing that IL-6 stimulates FTO protein levels in HuH7 cells expressing LepRb. Data are means ± SEM (n = 4/group).*p < 0.05 compared to untreated cells.

To examine the regulation of FTO expression by LepRb-STAT3 signalling pathway, we used immortalized HuH7 cells, as an in vitro model of hepatocytes. As they poorly respond to leptin treatment (Figure 1A), we transfected them with an expression vector coding for LepRb, and measured leptin-induced STAT3 phosphorylation to validate leptin action in LepRb-transfected HuH7 cells. Acute leptin treatment (5 and 15 minutes) did not significantly modify the Y705 phosphorylation of STAT3 in LepRb-transfected cells (Additional file 1: Figure S1A). However, LepRb overexpression induced STAT3 tyrosine phosphorylation (Y705) upon longer leptin stimulation (Figures 1A and B). Interestingly, LepRb overexpression per se induced FTO protein expression (Figures 1A and C), however 3-hour leptin treatment no further increased FTO expression in LepRb expressing HuH7 cells (Figures 1A and C). Since IL-6, like leptin, can activate STAT3 pathways [22], we further investigated whether IL-6 could also affect FTO expression in control HuH7 cells. Acute IL-6 treatment (10 ng/ml) had significant but weak effect on Y705 phosphorylation of STAT3 (Additonal file 1: Figure S1B). Indeed, IL-6 treatment induced Y705 phosphorylation of STAT3 in a time-dependent manner (Figure 2A) concomitantly inducing FTO protein expression with a maximal effect at 6 hours of treatment (Figure 2B, +70%, p < 0.05), suggesting that FTO could be a target gene of STAT3 in hepatocytes. Silencing of STAT3 expression using a specific siRNA (−50% of STAT3 mRNA levels, p < 0.001) blocked leptin-induced induction of FTO mRNA levels (leptin effect on FTO/TBP mRNA: -9% vs +51% in STAT3-silenced HuH7 cells or in control cells, respectively, p < 0.05), confirming that FTO is a target gene of STAT3.

### FTO regulates the LepRb-STAT3 signalling pathway in HuH7 cells

**Figure 3:**
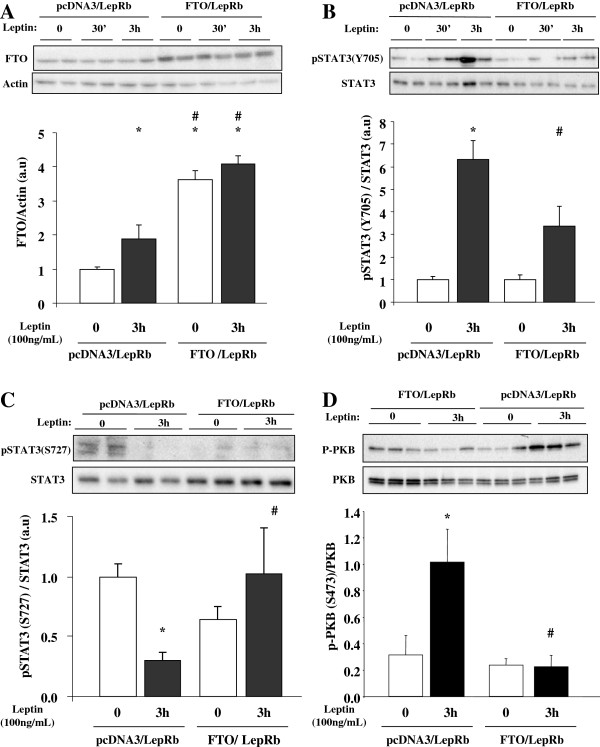
**FTO regulates the LepRb-STAT3 signalling pathway in co-transfected HuH7 cells.** HuH7 cells were co-transfecting with both LepRb and FTO vectors (empty vector as control) for 36 hours. Cells were then serum starved for 16 hours before three-hour leptin stimulation (100 ng/mL). **A)** Representative Western blots and quantitative analysis of FTO expression in co-transfected HuH7 cells, validating the FTO overexpression. **B)** Representative Western blots and quantitative analysis of pSTAT3(Y705) and total STAT3 in co-transfected HuH7 cells, showing that FTO represses the leptin-induced STAT3 tyrosine phosphorylation. **C)** Representative Western blots of pSTAT3(S727) and total STAT3 in co-transfected cells, showing that FTO represses the leptin-mediated reduction of serine phosphorylation of STAT3. **D)** Representative Western blots and quantitative analysis of pPKB(S473) and PKB proteins in co-tranfected HuH7 cells, showing that FTO represses the leptin-induced PKB phosphorylation. Data are means ± SEM (n = 3-6/group). *p < 0.05 compared to untreated control cells. #p < 0.05 compared to treated control cells.

To investigate the effects of FTO on the LepRb-STAT3 signalling pathway, we then co-expressed LepRb and FTO in HuH7 cells, and investigated leptin actions on STAT3 phosphorylations. The efficiency of the co-transfections was confirmed by the increased FTO protein levels (Figure 3A), as well as by the ability of leptin to phosphorylate STAT3 on Y705 (Figure 3B). Leptin significantly increased FTO protein expression in control co-transfected cells, but not in cells overexpressing FTO (Figure 3A). Importantly, FTO overexpression reduced Y705 STAT3 phosphorylation by leptin by 1.9 fold, compared to leptin-stimulated control co-transfected cells (Figure 3B). In addition, leptin response on S727 STAT3 phosphorylation was also affected by FTO overexpression, since leptin could not decrease S727 STAT3 phosphorylation in presence of FTO, as in control conditions (Figure 3C). These data indicate thus that FTO interacts with leptin-induced STAT3 phosphorylation both on tyrosine 705 and serine 727 residues in HuH7 cells. Another pathway regulated by LepRb is the phosphorylation of PKB. Then, we investigated whether FTO impact also on leptin-induced PKB phosphorylation (S473) in HuH7 cells. As shown on Figure 3D, leptin induced PKB phosphorylation in LepRb-transfected cells (3 fold, p < 0,005), and FTO overexpression blocked this regulation.

**Figure 4:**
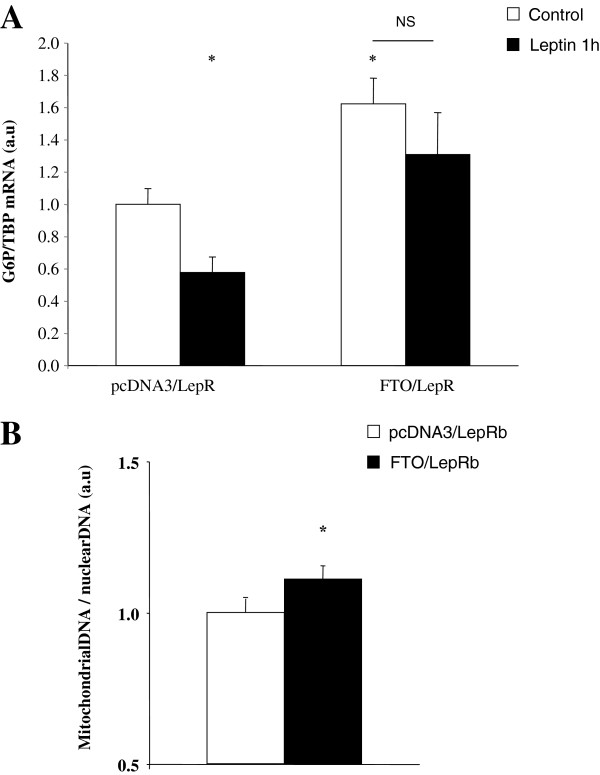
**FTO regulates downstream events of activated STAT3 in co-transfected HuH7 cells.** HuH7 cells were co-transfecting with both LepRb and FTO vectors (empty vector as control) for 36 hours. Cells were then serum starved for 16 hours before three-hour leptin stimulation (100 ng/mL). **A)** Effect of FTO overexpression on G6P mRNA levels of leptin-stimulated co-transfected HuH7cells, measured by real-time RT-PCR and expressed relative to untreated control co-transfected HuH7 cells. **B)** Effect of FTO overexpression on mtDNA amount of co-transfected HuH7 cells. Data are means ± SEM (n = 6/group). *p < 0.05 compared to untreated control cells **A)** or to mock transfected cells **B)**. G6P: Glucose 6 phosphatase. TBP: TATA box binding protein.

We further investigated the consequences of FTO overexpression on leptin-regulated downstream steps of STAT3. STAT3 tyrosine phosphorylation is required for dimer formation, nuclear translocation, and thus for the DNA binding activity of this transcriptional regulator [23]. Among STAT3-regulated genes, G6P gene is repressed by STAT3 in HuH-7 cells [19]. Thus, we measured FTO overexpression effect on G6P expression. As expected, one hour of leptin treatment repressed the transcription of G6P gene in control condition (Figure 4A). However, G6P mRNA levels were significantly increased in basal conditions of FTO overexpressing HuH7 cells and leptin-induced repression of G6P transcription was totally abolished (Figure 4A). On the other hand, S727 STAT3 phosphorylation has been shown to favor translocation of STAT3 to the mitochondria and to regulate mitochondrial activity [10]. Therefore, we investigated the consequence of FTO-mediated alteration of S727 STAT3 phosphorylation on cellular mitochondrial density in co-transfected HuH7 cells. As shown in Figure 4B, FTO expression significantly increased the mitochondrial DNA/nuclear DNA ratio in co-transfected HuH7 cells (p < 0.05). These finding suggest that FTO expression in liver cells could alter leptin-induced Y705 and S727 STAT3 phosphorylations impacting on metabolic gene expression and mitochondrial density.

### Disruption of STAT3 signalling in liver of mice overexpressing FTO

**Figure 5:**
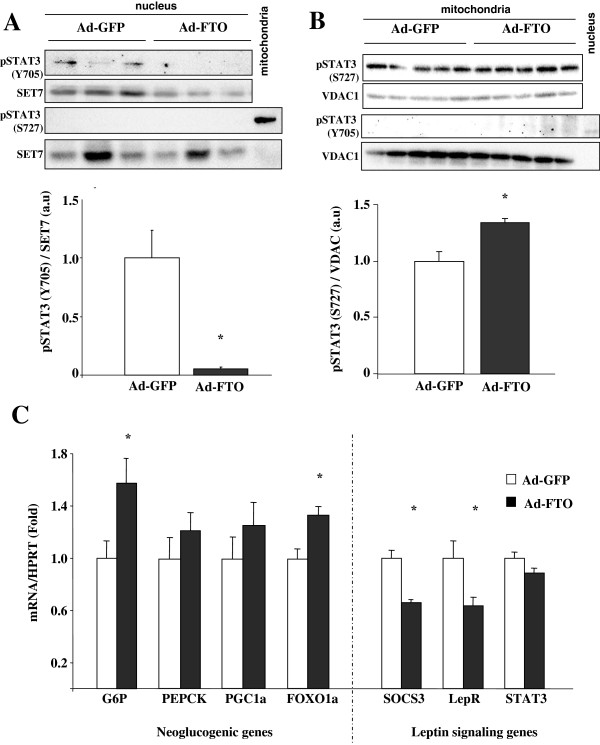
**Overexpression of FTO in liver of mice disruptsSTAT3 pathway.** FTO was overexpressed in liver of mice by adenoviral infection using a recombinant adenovirus encoding human FTO or GFP (as control) proteins for 10 days (2.10^8^ifu/g of body weight). **A)** Representative Western blots and quantitative analysis of pSTAT3(Y705) and SET7 proteins in nuclear fractions of liver infected with a recombinant adenovirus encoding human FTO or GFP (as control) proteins for 10 days. Controls with a marker of mitochondrial fractions illustrate that pS-STAT3 is nor present in nucleus fractions. **B)** Representative Western blots and quantitative analysis of pSTAT3(S727) and VDAC in mitochondrial fractions of infected liver. Controls with a marker of nucleus fractions illustrate that pY-STAT3 is nor present in mitochondrial fractions. **C)** mRNA levels of G6P, PEPCK, PGC1α and FOXO1α determined by real-time PCR in liver of Ad-GFP and Ad-FTO mice and expressed relative to Ad-GFP mice Data are means ± SEM (n = 4/group for **A** and n = 6/group for **B**). *p < 0.05 compared to Ad-GFP mice.

To determine the physiological relevance of these observations in vivo, we investigated the impact of liver-targeted FTO expression in mice. We infected male C57BL/6 mice by retroorbital injections of recombinant adenovirus coding for FTO or GFP (as control), and followed STAT3 phosphorylation in both nuclear and mitochondrial liver fractions. As shown in Additional file 1: Figure 2, under this experimental setting, FTO was specifically overexpressed in liver, since no change of FTO mRNA levels was observed in epididymal adipose tissue and hypothalamus of Ad-FTO infected mice compared to Ad-GFP mice. In agreement with in vitro data, we found a decreased content of nuclear STAT3 phosphorylated on Y705 (normalized by the nuclear protein SET7) in nuclear fractions of FTO-infected mice compared to GFP-infected mice (Figure 5A, -94%, p < 0.01). Moreover, we found a significant increase of S727 STAT3 phosphorylation (normalized by VDAC1 protein) in mitochondrial fractions of FTO-infected mice compared to GFP-infected mice (Figure 5B, +34%, p < 0,01).

### FTO disrupts STAT3 actions in liver

**Figure 6:**
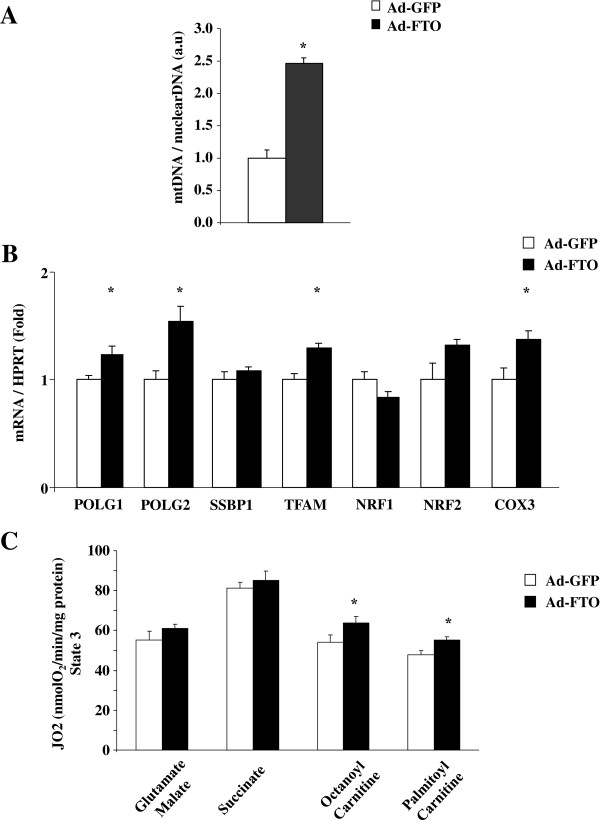
**FTO overexpression in liver of mice regulates mitochondrial density and funtion.** Liver of mice were infected with recombinant adenovirus encoding human FTO or GFP (control) proteins for 10 days (2.10^8^ifu/g of body weight). **A)** Effect of FTO on mtDNA quantity, calculated as the ratio of COX1 to cyclophilin A DNA levels, and measured by real-time PCR in the liver of infected mice. **B)** Effect of FTO on the mRNA levels of POLG1, POLG2, SSBP1, TFAM, NRF1, NRF2, COX3 measured by real-time PCR in the liver of Ad-GFP and Ad-FTO mice. **C)** Effect of FTO on oxygen consumption of mitochondria isolated from liver of Ad-GFP and Ad-FTO mice. Data are means ± SEM (n = 5/group). *p < 0.05 compared to GFP infected mice.

Then we measured the effects of FTO overexpression on downstream events of activated STAT3 in mice liver. In agreement with a decreased content of Y705 STAT3 phosphorylation upon FTO overexpression, we found a decrease of SOCS3 mRNA levels and an increase of G6P expression in liver of Ad-FTO mice (Figure 5C). Furthermore, the mRNA levels of the transcription factor FOXO1α was significantly increased whereas only a tendency was observed for PEPCK and PGC1α, following FTO overexpression (Figure 5C), suggesting that FTO may participate in the control of neoglucogenic genes expression by interacting with STAT3 in liver. We also found that the mtDNA/nuclear DNA ratio was markedly increased (2.5 fold, p < 0.05) in liver of Ad-FTO mice (Figure 6A) in agreement with FTO-induced increase of S727 STAT3 phosphorylation. Furthermore, POLG1 and POLG2 and TFAM, key genes implicated in mitochondrial replication were also induced in liver of Ad-FTO mice (Figure 6B). Moreover, the mRNA levels of the mitochondria-encoded gene COX3 were increased in liver of Ad-FTO mice (Figure 6B). We then measured oxygen consumption by isolated mitochondria from liver of infected mice. As shown in Figure 6C, FTO overexpression significantly increased oxygen consumption stimulated by either octanoyl- or palmitoyl-CoA (+18,2% and +15,5%, respectively, p < 0,05), whereas the respiration under glutamate/malate or succinate substrates were not significantly modified. Altogether, these results confirmed in vivo that FTO may regulate both hepatic neoglucogenenic gene expression and oxidative metabolism by interacting with the STAT3 signaling pathways.

### FTO overexpression alters leptin action and glucose homeostasis in mice

**Figure 7:**
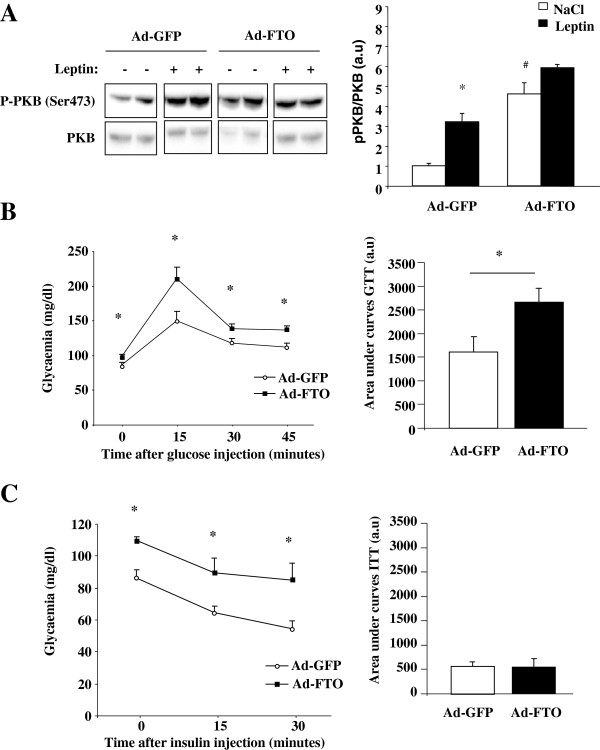
**FTO overexpression in liver of mice affects leptin action and glucose homeostasis.** Liver of mice were infected with recombinant adenovirus encoding human FTO or GFP (control) proteins for 10 days (2.10^8^ifu/g of body weight). **A)** After an overnight fasting, infected mice were treated by ip injection with leptin (1 μg/g) for 30 minutes. Representative Western blots and quantitative analysis of pPKB(S473) and PKB proteins in liver of leptin-treated infected mice. Note that four parts of a same gel were shown. **B)** Glucose tolerance test performed after 6 hours of fasting on infected mice and corresponding quantitative analysis of area under curves. **B)** Insulin tolerance test performed after 6 hours of fasting on infected mice and corresponding quantitative analysis of area under curves. Data are means ± SEM (n = 3/group for **A** and n = 8/group for **B-C)**. *p < 0.05 compared to GFP infected mice.

We found that Ad-FTO mice have increased circulating leptin concentration in the fasting state when compared to Ad-GFP mice (Table 1), suggesting compensatory mechanism against a state of leptino-resistance. In agreement, the mRNA levels of LepR and SOCS3, but not STAT3, were decreased in liver overexpressing FTO (Figure 5B). To verify whether leptin action was reduced in vivo, we investigated the effect of FTO on leptin-induced PKB phosphorylation. For that, we infused leptin to fasted infected C57BL/6 mice and measured the repercussion on the phosphorylation of PKB 30 minutes after treatment. As shown on Figure 7A, leptin induced a 3-fold induction of S473 phosphorylation of PKB in Ad-GFP mice and this effect was lost following FTO overexpression, in agreement with in vitro data. Although this was due in part to an increase of basal PKB phosphorylation (Figure 7A), this result indicated that FTO overexpression was able to prevent leptin action in vivo in mice liver.

**Table 1:** Metabolic paramaters of Ad-GFP and Ad-FTO mice after an overnight fasting

	**Ad-GFP mice**	**Ad-FTO mice**	**p value**
Body weight (g)	22.5 ± 0.5	23.8 ± 0.5	NS
Liver weight (g)	1.6 ± 0.06	1.5 ± 0.06	NS
Fasting glycemia (mg/dl)	86 ± 5	110 ± 3	0.002
Fasting insulinemia (ng/ml)	0.22 ± 0.03	0.50 ± 0.01	0.02
Fasting leptinemia (ng/ml)	0.5 ± 0.1	13 ± 0.3	0.03

FTO was overexpressed in liver of mice by adenoviral infection using a recombinant adenovirus encoding human FTO or GFP (as control) proteins for 10 days (2.10^8^ifu/g of body weight). Blood was removed by reorbital punction on isofluran-anesthesized mice, after an overnight fasting. Data are means ± SEM (n = 8/group).

We then verified whether FTO-mediated disruption of leptin-STAT3 signalling could alter glucose homeostasis in mice. We found that both fasting glycemia and insulinemia were significantly higher in Ad-FTO mice compared to Ad-GFP mice (Table 1). Moreover, glucose tolerance test revealed that Ad-FTO mice were glucose intolerant (Figure 7B). However, despite hyperglycemia all along the test, the response of glycemia to insulin injection (insulin tolerance test) was similar between both mice (Figure 7C), suggesting that FTO overexpression in the liver did not impair peripheral insulin sensitivity.

## Discussion

The regulation and functions of FTO in liver are largely unknown. In this study we report a new role of FTO as a regulator of leptin-STAT3 pathway in hepatocytes using both in vitro and in vivo approches. We demonstrate that FTO overexpression disturbs LepRb-STAT3 signalling pathway with both a reduction of leptin-induced Y705 phosphorylation and an induction of S727 phosphorylation of STAT3, which is normally repressed by leptin. The effect on tyrosine phosphorylation affects STAT3 translocation to the nucleus and regulation of leptin target genes. The effect on STAT3 S727 phosphorylation favors its translocation to the mitochondria and increases mitochondria density and function. As a consequence of this dual effect on leptin-induced STAT3 phosphorylation, FTO alters leptin action and glucose homeostasis in liver.

Leptin, as other cytokines like IL-6, activate the tyrosine phosphorylation of STAT3 via specific membrane receptors, inducing its translocation into the nucleus where it regulates gene expression. Whereas LepRb is most abundantly expressed in the brain, it is also present in peripheral tissue, in particular in liver [8,24,25]. A previous study has challenged the presence of functional LepRb in hepatocytes [26], whereas other studies state that leptin was active on hepatocytes [27,28]. In our study, we demonstratedd that leptin has not only an action on LepRb-expressing HuH7 cells and in mice liver, but also in rat primary hepatocytes (Additional file 1: Figure 3A and B), validating that leptin has really a metabolic role in hepatocytes. Nevertheless, we observed that rather long treatment with leptin or IL-6 are required to activate the LepRb-STAT3 pathway. Consequently, we cannot exclude that an indirect or secondary effect in HuH7 cells, such as the production and secretion of other factors, participate to the leptin/IL-6-mediated activation of STAT3 in 3 hour-treated HuH7 cells. Thus, we propose that leptin likely acts on hepatocytes through both direct and indirect mechanisms. In addition to increase STAT3 tyrosine phosphorylation, we further demonstrated that leptin reduced the serine phosphorylation of STAT3 in LepRb-transfected cells HuH7 cells. This data are in agreement with a recent study demonstrating that leptin receptor-free tumor cells display increased STAT3 serine phosphorylation on residue S727, and preserved mitochondrial function [29]. As both phosphorylation sites on STAT3 are in close proximity (Y705 and S727), it is possible that pS727-STAT3 enhances the dephosphorylation of pY705, as recently suggested [11], or that the phosphorylation of one residue hinder the phosphorylation of the other ones. Consequently, we cannot exclude that the reduction of pS-STAT3 phosphorylation in response to leptin was a consequence of the increase of leptin-mediated pY-STAT3 phosphorylation in HuH7 cells.

We found in HuH7 cells that leptin and IL-6 induce FTO expression and that silencing of STAT3 inhibits leptin-mediated regulation of FTO, suggesting that FTO gene can be directly controlled by STAT3. This finding confirms in liver cells previous studies demonstrating that FTO expression could be regulated by nutritional status via LepRb-STAT3 signalling pathway in hypothalamus [12]. It is also in line with the observation that FTO mRNA levels were positively correlated with G6P and PEPCK expression in liver [20].

More interestingly, we demonstrated both in vitro and in vivo that FTO impacts LepR-STAT3 pathway via a fine control of STAT3 phosphorylation, as well as PKB pathway. Concerning STAT3, FTO overexpression in liver of mice decreases Y705 STAT3 phosphorylation in nucleus associated with an increased mitochondrial S727 STAT3 phosphorylation and reduced leptin-regulated phosphorylation of STAT3 on both sites in LepRb expressing HuH7 cells. This suggest that FTO favors STAT3 mitochondrial translocation at the expense of nuclear localization in liver, leading to an upregulation of neoglucogenic genes and to an increase of mitochondrial density and function in liver of mice overexpressing FTO. Consequently, these data are in agreement with recent studies showing that the preferential localization of S727 phosphorylated STAT3 into mitochondria is associated with an increase of mitochondria respiration [10,30], and further suggest that FTO may regulate energy metabolism in the liver likely though mitochondrial STAT3 localization. These effects have detrimental in vivo consequences both on leptin action and glucose homeostasis. Indeed, liver FTO overexpression induced an inhibition of leptin-induced PKB phosphorylation, a down-regulation of leptin-regulated genes (SOCS3, LepR, G6P and FOXO1). Furthermore, Ad-FTO mice developed hyperleptinemia, indicating that FTO inhibits leptin action, and mimic a state of leptin resistance. Interestingly, mitochondrial respiration is increased in liver of leptin deficient ob/ob mice [31], and leptin was reported to reduce hepatic metabolism in ob/ob mice via changes in mitochondria function, structure, and protein levels [32], suggesting that the mitochondrial effects of FTO could be a consequence of FTO-mediated inhibition of leptin action. In addition, Ad-FTO mice develop hyperglycemia, hyperinsulinemia and glucose intolerance, pointing thus a novel important role of FTO in the regulation of glucose metabolism. These data are in agreement with previous data showing that FTO-deficient mice show alterations of energy homeostasis [6] and that hepatic FTO expression is regulated by metabolic state [20]. Furthermore, the data are supported by STAT3 manipulation models of mice showing that liver specific knock-out induces neoglucogenic gene expression and hepatic insulin resistance [18], whereas liver specific overexpression of STAT3 reduces glycemia, insulinemia and neoglucogenic gene expression [19]. FTO may therefore be a novel regulator of STAT3 metabolic actions in liver cells.

The exact function of FTO remains unknown. Recombinant FTO protein catalyses the Fe(II)-and 2-oxoglutarate-dependant demethylation of RNA [14] and N6-methyl-adenosine [33]. This demethylation could stabilize the target mRNA and increase its expresion level. Recently, Karra E et al. reported that both FTO overexpression and FTO obesity-risk alleles (AA) were associated with reduced ghrelin mRNA N6-methyladenosine methylation and increased ghrelin expression [34], confirming in vivo a role of FTO in methylation process. In our study, the mechanisms by which FTO controls the LepRb-STAT3 signalling pathway and leptin action is unknown, and further studies are required to determine whether methylation processes are implicated. We can only speculate that if it occurs, it probably does not affect LepR, SOCS3 or STAT3 mRNAs directly since both LepR and SOCS3 expression is down-regulated, whereas STAT3 expression is not affected by FTO overexpression. Nevertheless, our data indicate that FTO could participate to metabolic regulations in liver. In aggreement, FTO expression is increased in liver of a rat model of nonalcoholic fatty liver disease [35], and FTO overexpression increased oxydative stress and lipogenesis in L02 cells [35] and myotubes [3]. In addition, as leptin was shown to regulate immunoreaction in liver, playing a critical role to hyperreactivity against endotoxin during NASH progression [36], further studies are required to determine whether FTO could impact this pathway. Finally, it should be noted that S727 STAT3 phosphorylation is needed for constitutive activation of STAT3 and cell invasion in various human cancers [37,38]. Therefore, interaction between FTO and STAT3 may suggest potential implication of FTO in cancer development, as it has already been suggested [39].

## Conclusions

**Figure 8:**
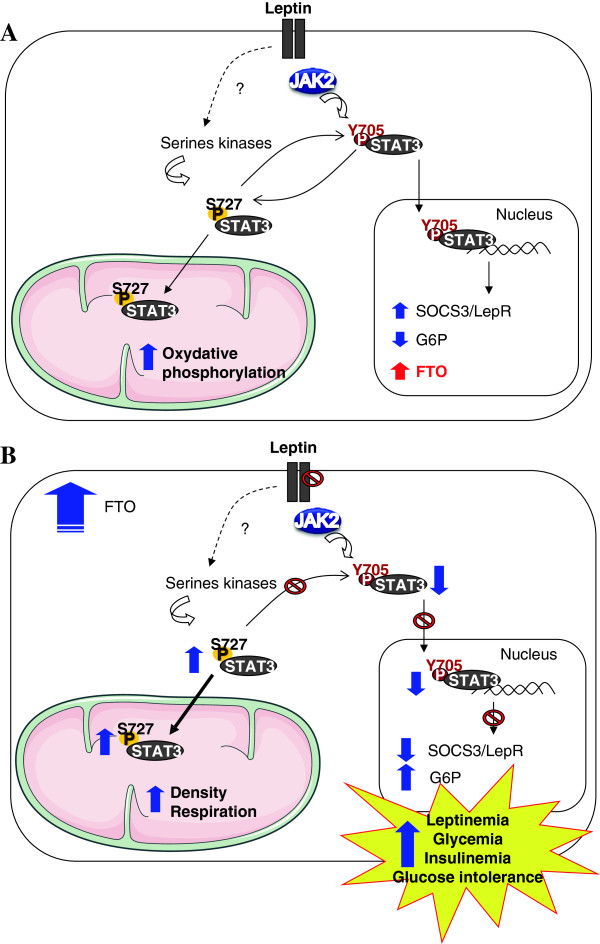
**Model of FTO regulation of leptin action and glucose homeostasis through the LepRb-STAT3 signaling pathway. A)** The binding of leptin to its receptor triggers the activation of JAK2 and the phosphorylation of STAT3 on a tyrosine residue (Y705). This modification causes the STAT3 protein to relocate to the nucleus, where as a dimer, it regulates gene expression (upregulation of both SOCS3 and LepR and reduction of G6P). Our data further suggest that FTO could be a STAT3-upregulated gene in hepatocytes. **B)** FTO overexpression reduces Y705 STAT3 phosphorylation, reducing its nuclear translocation and leading to a downregulation of both SOCS3 and LepRb and an upregulation of G6P mRNA levels. Just to opposite, FTO overexpression induces S727 phosphorylation of STAT3, favorizing its mitochondrial localization, where it induces mitochondrial density and function. All these effects are associated in vivo with hyperleptinemia, hyperglycemia, hyperleptinemia and glucose intolerance. Altogether, these data indicate that FTO controls leptin action and glucose metabolism in liver.

In conclusion, our study highlights a new function of hepatic FTO in the regulation of leptin action and the control energy metabolism via interactions with STAT3 signalling (Figure 8). Actions of FTO could involve a recruitment of S727-phosporylated STAT3 into mitochondria, at the expense of nuclear localization, impacting subsequently both mitochondrial oxidative metabolism and neoglucogenic gene expression. Because it is well known that leptin and STAT3 are important players of metabolic diseases, our results suggest that the role of FTO in the liver should be taken into consideration for the understanding of the metabolic regulations and that FTO polymorphisms could contribute to metabolic complications in obesity and diabetes.

## Methods

### Mouse

Animal studies were performed in accordance with the French guidelines for the care and used of animals and were approved by our regional ethic committee. Eight week-old male C57BL/6 mice (Harlan) were housed in controlled environment. Recombinant adenoviruses encoding for FTO or GFP proteins (Ad-FTO and Ad-GFP, respectively) were injected retroorbitally in mice (2.10^8^ infection forming units (ifu)/g of body weight), in order to over-express proteins specifically in liver. Glucose and insulin tolerance test were done respectively on day 7 and 9 after adenoviral infection. During these tests, glycemia were followed every 15 minutes after intraperitoneally injection of glucose (2 mg/g of body weight) or insulin (0.75 mUI/g of body weight). On day 10, blood was collected from animals by retroorbital punction under isofluran anesthesia, and mice were killed by cervical dislocation. Blood glucose levels were measured using a glucometer (Roche Diagnostics). Serum levels of insulin and leptin were determined using murine ELISA kits (ALPCO).

### Primary hepatocytes

Rat primary hepatocytes were isolated in the presence of collagenase according to the method of Berry and Friend [40], modified by Groen et al. [41].

### Constructions

The cDNA sequence encoding full-length human FTO was generated as previously described [3]. Recombinant adenoviral genome encoding human FTO was generated by homologous recombination and amplified as described previously [3]. The plasmid encoding an HA-tagged murine LepRb was generated as previously described [42].

### Cell culture and transfection

HuH7 cells were grown in Dulbecco modified Eagle’s medium (PAA) supplemented with 10% fetal bovine serum. Cells were transfected with 1 μg expression plasmids for the FTO gene or for the LepRbgene (pcDNA3-FTO and pCIneo-LepRb, respectively), using EXGEN 500 transfecting reagent (Euromedex). In cotransfection experiments, cells received at the same time 1 μg of both vectors. An empty vector was used as control in each experiment. HuH7 cells were then used for treatments 48 hours post-transfection. Treatments included leptin (Labomics, 100 ng/ml) and IL-6 (Sigma, 10 ng/mL) incubations after a 16 hour serum depletion.

### Total RNA preparation and quantification of messenger RNAs

Total RNA from tissues or cell cultures were purified using the TRI Reagent Solution (Sigma). mRNA levels were measured by reverse-transcription followed by real time quantitative PCR using a Rotor-Gene 6000 (Corbett Research), as previously described [3]. Primers are listed in Additional file 2: Table S1. Values were normalized using HPRT or TBP, which were similar among conditions.

### Western blot analysis

Tissues lysis and both separation and revelation of proteins were performed as described previously [3]. The primary antibodies used for protein detection are: STAT3 (Cell Signaling, #4904), Phospho-STAT3 (Tyr705) (Cell signaling, #9145), Phospho-STAT3 (Ser727) (Cell signaling, #9134), FTO (Abcam, ab65366), Actin (Sigma, A5060), SET7/9 (Santa Cruz, sc-56774), VDAC1/Porin (Abcam, ab14734).

### Subcellular fractionation

Liver was homogenised in isolation buffer (Mannitol 210 mM, Saccharose 70 mM, Tris 50 mM, EDTA 10 mM and BSA 0.5%, pH = 7.4) using a teflon pestle, and centrifuged 10 minutes at 800 g. The pellet was kept for further nuclei isolation whereas the supernatant was centrifuged 10 minutes at 8000 g for mitochondria isolation, as previouly described [43]. The pellet of mitochondria was resuspended in isolation buffer, centrifuged a second time 10 minutes at 8000 g and resuspended in isolation buffer. For nuclei isolation, the pellet from the first centrifugation was resuspended in a hypertonic buffer (Hepes 10 mM, NaCl 0.42 M, MgCl2 1.5 mM, Glycerol 2.5%, EDTA 1 mM, EGTA 1 mM, DTT 1 mM, protease inhibitors cocktail 1X, pH = 7.4) and centrifuged for 30 min at 100 000 g in order to get nuclear extract in supernatant.

### Measurement of mitochondrial respiration on isolated mitochondria

Mitochondrial respiration rates were measured at 30°C on freshly isolated liver mitochondria using a closed-thermostated oxygraph (Stratkelvin, UK). Different substrates were used: glutamate 5 mM + malate 2.5 mM as complex 1 substrates; succinate 5 mM + rotenone 5 μM as a complex 2 substrates with inhibition of complex 1 by rotenone; octanoyl-carnitine (110 μM) or palmitoyl-carnitine (55 μM) in presence of 1 mM malate, as β-oxydation substrates. State 3 was measured in the presence of respiratory substrates after the addition of 1 mM ADP and state 4 was measured after the addition of oligomycin (2 μg/mL).

### Mitochondrial DNA analysis

The extraction of total DNA and the measurement of mitochondrial DNA (mtDNA) content by real time PCR was performed as previously described [43].

### Statistical analyses

All data are represented by means ± SEM. Statistical significance was determined using student unpaired t test. The threshold for significance was set at p < 0.05.

## Abbreviations

COX1/3: Cytochrome c oxidase subunit 1, 3; FOXO1α: Forkhead box O1 alpha; FTO: Fat mass and obesity associated gene; G6P: Glucose 6 phosphatase; GFP: Green fluorescent protein; HPRT: Hypoxanthine guanine phosphoribosyl transferase; JAK2: Janus kinase 2; LepRb: Leptin receptor long isoform; mtDNA: Mitochondrial DNA; NRF1: Nuclear respiratory factor 1; NRF2: Nuclear respiratory factor 2; PEPCK: Phosphoenol pyruvate carboxykinase; PGC1α: Peroxisome proliferator-activated receptor gamma, coactivator 1 alpha; POLG1/2: DNA Polymerase subunit gamma 1, 2; SET7: Methyltransferase; SOCS: Suppressor of signaling cytokine 3; STAT3: Signal transducer and activator of transcription 3; SSBP1: Single-stranded DNA-binding protein 1; TBP: Tata box binding protein; TFAM: Transcription factor A, mitochondrial.

## Competing interests

The authors declare that there is no duality of interest associated with this manuscript.

## Authors’ contributions

AB, GV, JR, and HV contributed to the conception and design of the study. AB, GV and MAC contributed to the acquisition of data. AB, GV and JR contributed to analysis and interpretation of the data. All authors contributed to drafting the article or critically revising its intelectual content, and all authors approved the final version. All authors read and approved the final manuscript.

## Supplementary Material

Additional file 1: Figure S1 Acute leptin and IL-6 treatments on pY-STAT3 phosphorylation in HuH7 cells. Figure S2. Validation of the specific overexpression of FTO in liver of infected mice. Figure S3. Effect of leptin on pY-STAT3 phosphorylation and G6P expression in rat primary Hepatocytes. Click here for file

Additional file 2: Table S1 Sequences of primer. Click here for file
