# Supplementary material for: FTO contributes to hepatic metabolism regulation through regulation of leptin action and STAT3 signalling in liver
Source: Cell Commun Signal. 2014 Jan 10;12:4. doi: 10.1186/1478-811X-12-4 (PMC3896784; doi:10.1186/1478-811X-12-4)
Supplement: Additional file 1: Figure S1 — Acute leptin and IL-6 treatments on pY-STAT3 phosphorylation in HuH7 cells. Figure S2. Validation of the specific overexpression of FTO in liver of infected mice. Figure S3. Effect of leptin on pY-STAT3 phosphorylation and G6P expression in rat primary Hepatocytes. [file 1478-811X-12-4-S1.ppt]

## Slide 1
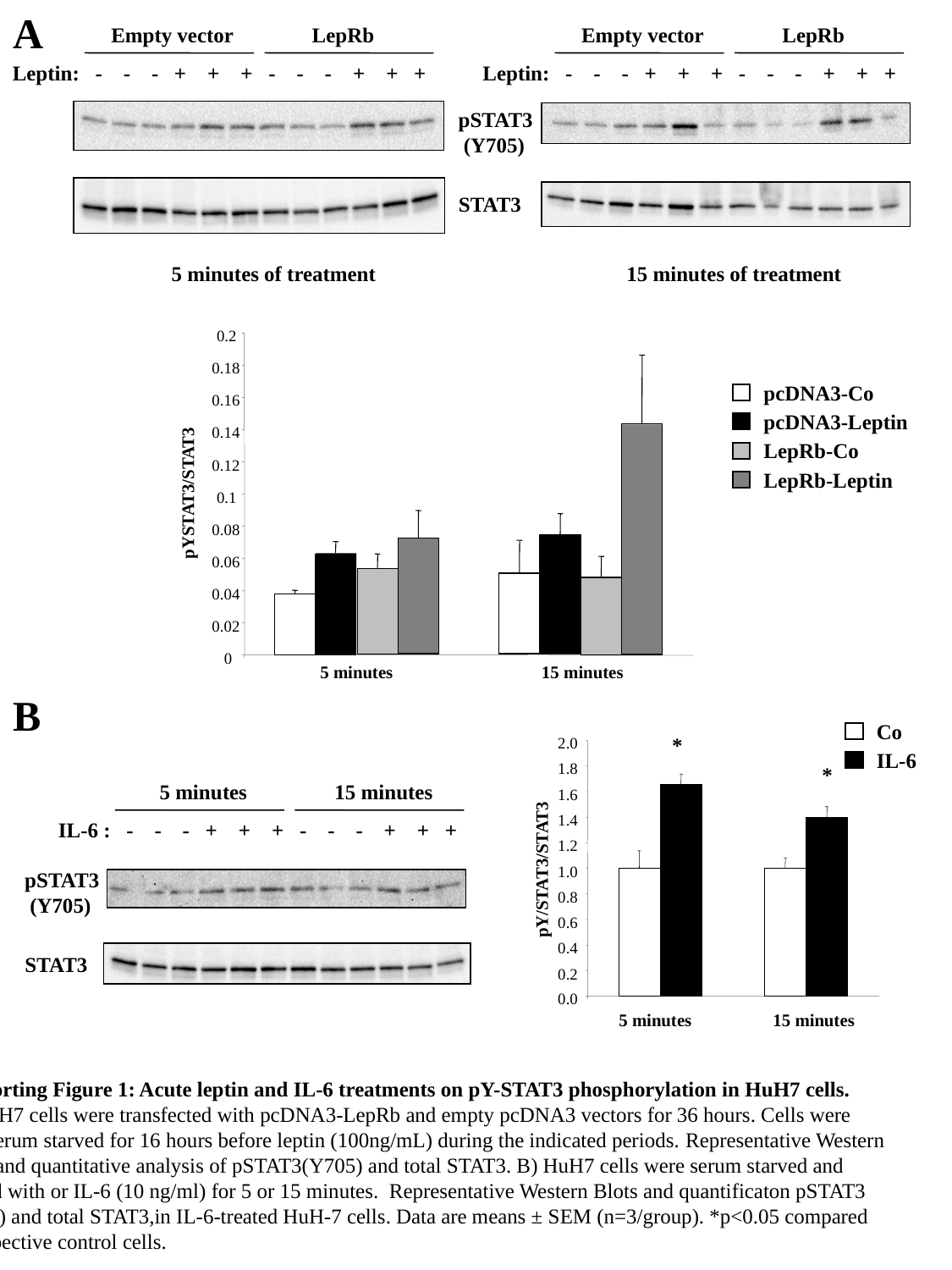

A
 Empty vector
 LepRb
 Empty vector
 LepRb
Leptin: - - - + + + - - - + + +
Leptin: - - - + + + - - - + + +
pSTAT3
 (Y705)
STAT3
5 minutes of treatment
15 minutes of treatment
0.2
0.18
pcDNA3-Co
0.16
pcDNA3-Leptin
0.14
LepRb-Co
0.12
LepRb-Leptin
pYSTAT3/STAT3
0.1
0.08
0.06
0.04
0.02
0
5 minutes
15 minutes
B
Co
*
2.0
IL-6
*
1.8
 5 minutes
 15 minutes
1.6
IL-6 : - - - + + + - - - + + +
1.4
1.2
pY/STAT3/STAT3
pSTAT3
 (Y705)
1.0
0.8
0.6
0.4
STAT3
0.2
0.0
 5 minutes
 15 minutes
Supporting Figure 1: Acute leptin and IL-6 treatments on pY-STAT3 phosphorylation in HuH7 cells.
A) HuH7 cells were transfected with pcDNA3-LepRb and empty pcDNA3 vectors for 36 hours. Cells were
then serum starved for 16 hours before leptin (100ng/mL) during the indicated periods. Representative Western
Blots and quantitative analysis of pSTAT3(Y705) and total STAT3. B) HuH7 cells were serum starved and
treated with or IL-6 (10 ng/ml) for 5 or 15 minutes. Representative Western Blots and quantificaton pSTAT3
(Y705) and total STAT3,in IL-6-treated HuH-7 cells. Data are means ± SEM (n=3/group). *p<0.05 compared
to respective control cells.

## Slide 2
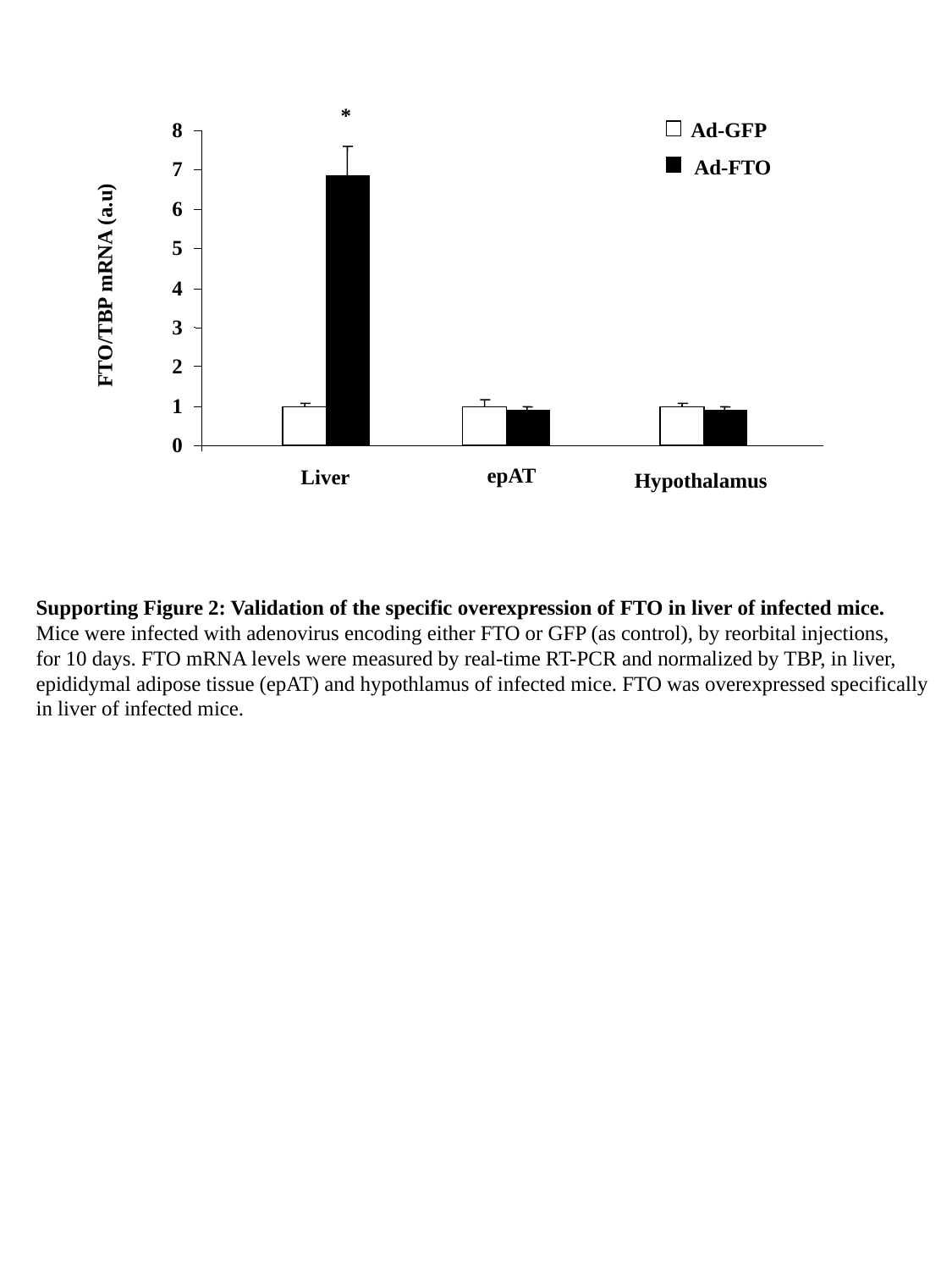

*
8
Ad-GFP
Ad-FTO
7
6
5
FTO/TBP mRNA (a.u)
4
3
2
1
0
epAT
Liver
Hypothalamus
Supporting Figure 2: Validation of the specific overexpression of FTO in liver of infected mice.
Mice were infected with adenovirus encoding either FTO or GFP (as control), by reorbital injections,
for 10 days. FTO mRNA levels were measured by real-time RT-PCR and normalized by TBP, in liver,
epididymal adipose tissue (epAT) and hypothlamus of infected mice. FTO was overexpressed specifically
in liver of infected mice.

## Slide 3
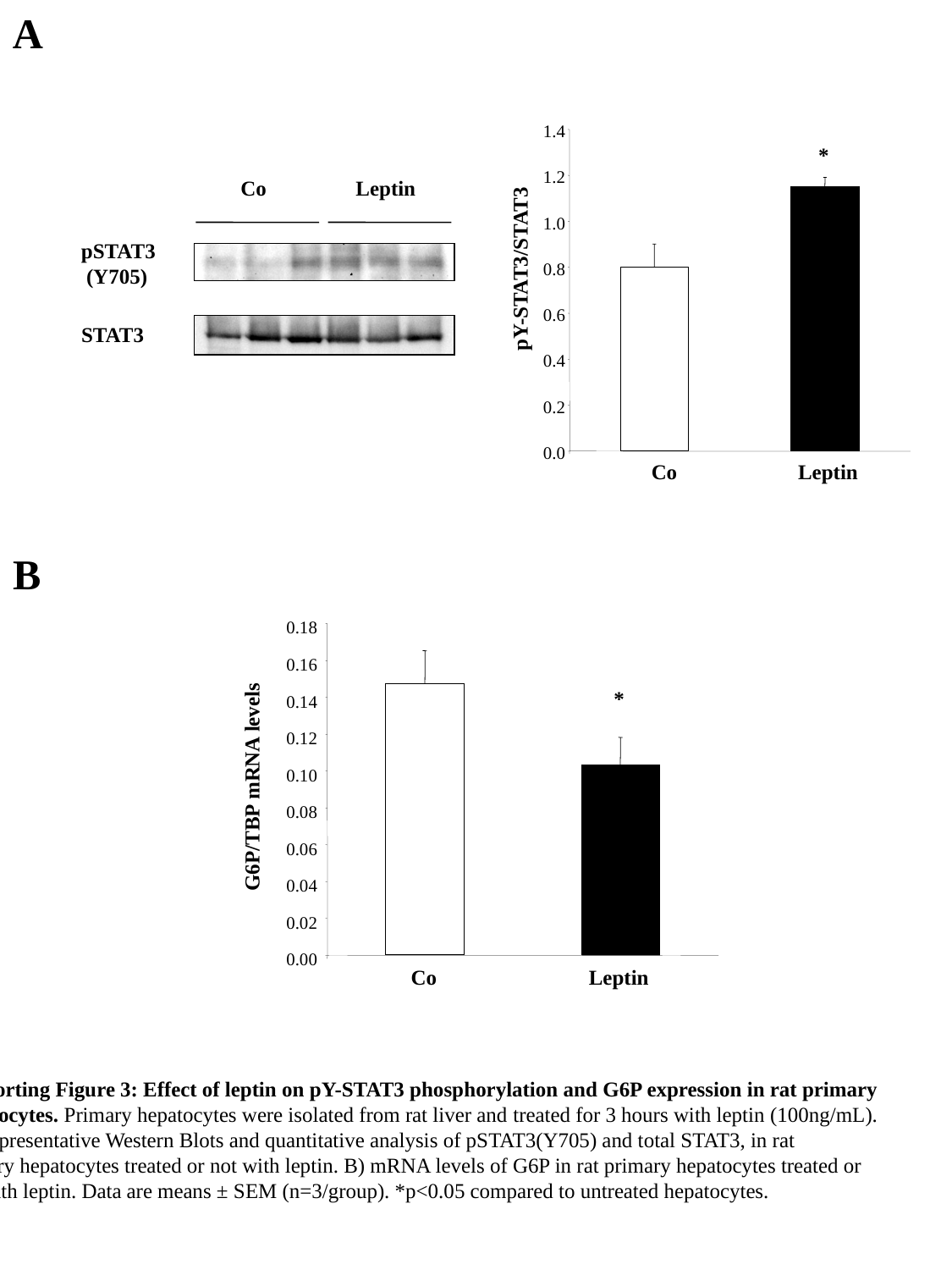

A
1.4
1.2
1.0
pY-STAT3/STAT3
0.8
0.6
0.4
0.2
0.0
Co
Leptin
*
 Co
 Leptin
pSTAT3
 (Y705)
STAT3
B
0.18
0.16
*
0.14
0.12
0.10
G6P/TBP mRNA levels
0.08
0.06
0.04
0.02
0.00
Co
Leptin
Supporting Figure 3: Effect of leptin on pY-STAT3 phosphorylation and G6P expression in rat primary
hepatocytes. Primary hepatocytes were isolated from rat liver and treated for 3 hours with leptin (100ng/mL).
A) Representative Western Blots and quantitative analysis of pSTAT3(Y705) and total STAT3, in rat
primary hepatocytes treated or not with leptin. B) mRNA levels of G6P in rat primary hepatocytes treated or
not with leptin. Data are means ± SEM (n=3/group). *p<0.05 compared to untreated hepatocytes.
