# Supplementary material for: FTO contributes to hepatic metabolism regulation through regulation of leptin action and STAT3 signalling in liver
Source: Cell Commun Signal. 2014 Jan 10;12:4. doi: 10.1186/1478-811X-12-4 (PMC3896784; doi:10.1186/1478-811X-12-4)
Supplement: Additional file 2: Table S1 — Sequences of primer. [file 1478-811X-12-4-S2.doc]

**Supplementary Table 1: Sequences of primers**

Primers for nuclear and mitochondrial DNA quantification

COX1 – S: ACTATACTACTACTAACAGACCG

COX1 – AS: GGTTCTTTTTTTCCGGAGTA

Cyclophilin A – S: ACACGCCATAATGGCACTGG

Cyclophilin A – AS: CAGTCTTGGCAGTGCAGAT

Primers for mRNA quantification

HPRT – S: TTG-CTG-ACC-TGC-TGG-ATT-AC

HPRT – AS: AGT-TGA-GAG-ATC-ATC-TCC-AC

FOXO1a – S: AGA-TCT-ACG-AGT-GGA-TGG-TG

FOXO1a – AS: GGA-CAG-ATT6GTG-GCG-AAT-TG

FTO – S: GAG-TTT-GAG-TGG-CTG-AGG-CA

FTO – AS: GGG-AGC-CCC-TCT-CTT-TTA-AC

G6P – S: TTACCAAGACTCCCAGGACTG

G6P – AS: GAGCTGTTGCTGTAGTAGTC

LepR – S: CAC-AAC-CGA-TGA-CTC-CTT-TC

LepR – AS: CAT-CCA-GCA-CTC-TAT-GTC-C

NRF1 – S: GCT-GCT-GCT-GTG-GCA-ACA-GG

NRF1 – AS: TTG-GGT-TTG-GAG-GGT-GAG-AT

NRF2 – S: CCA-AGT-CCT-GCA-TTG-GGT-GG

NRF2 – AS: GCA-AAA-ACT-GCC-ATA-GTT-GG

PEPCK – S: CCCGGACCGCAGAGAGATCATC

PEPCK – AS: TCCCACAGGCACTAGGGAAGGC

PGC1α – S: TCC-TCT-GAC-CCC-AGA-GTC-AC

PGC1α – AS: CTT-GGT-TGG-CTT-TAT-GAG-GAG-G

POLG1 – S: GCG-GCT-GGT-GGA-AGA-GCG-TT

POLG1 – AS: GAG-ATG-GCC-ATG-TGC-ATG-CTC

POLG2 – S: CAC-TAT-GTT-AAT-TGC-CTG-G

POLG2 – AS: CCA-GAA-ATC-AAG-CCA-CTG-G

SOCS3 – S: CCA-CGG-AAC-CCT-CGT-CCG-AAG-T

SOCS3 – AS: GTA-GTA-AGC-TCT-CTT-GGG-GG

SSBP1 – S: CGT-CAG-TTT-GTA-AGA-CAT-GAG

SSBP1 – AS: CCC-CTG-ATC-GCC-ACA-TCT-C

STAT3 – S: CAT-GGA-GGA-GTC-TAA-CAA-CG

STAT3 – AS: ATC-AGG-TGC-AGC-TCC-TCA-GTC

TBP – S: TGG-TGT-GCA-CAG-GAG-CCA-AG

TBP – AS: TTC-ACA-TCA-CAG-CTC-CCC-AC

TFAM – S: GCT TGG AAA ACC AAA AAG AC

TFAM – AS: CCC AAG ACT TCA TTT CAT T
